# Oxygen in Red Blood Cell Concentrates: Influence of Donors’ Characteristics and Blood Processing

**DOI:** 10.3389/fphys.2020.616457

**Published:** 2020-12-23

**Authors:** Manon Bardyn, Agathe Martin, Nora Dögnitz, Mélanie Abonnenc, Andrew Dunham, Tatsuro Yoshida, Michel Prudent

**Affiliations:** ^1^Laboratoire de Recherche sur les Produits Sanguins, Transfusion Interrégionale CRS, Epalinges, Switzerland; ^2^Laboratoire de Préparation Cellulaire et d’Analyses, Transfusion Interrégionale CRS, Epalinges, Switzerland; ^3^Département Approvisionnement Produits Sanguins, Transfusion Interrégionale CRS, Bern, Switzerland; ^4^Hemanext Inc., Lexington, MA, United States; ^5^Centre de Transfusion Sanguine, Faculté de Biologie et de Médecine, Université de Lausanne, Lausanne, Switzerland

**Keywords:** oxygen saturation (sO_2_), resonance Raman (RR) spectroscopy, red blood cell, red blood cell concentrate, donor variation, donors’ characteristics, blood processing, transfusion medicine

## Abstract

**Objective:** Unexpectedly wide distribution (<10 to >90%) of hemoglobin oxygen saturation (sO_2_) within red cell concentrates (RCCs) has recently been observed. Causes of such variability are not yet completely explained whereas the roles of oxygen and oxidative lesions during the storage of RCCs are known. The objectives of the present study are to characterize sO_2_ distribution in RCCs produced in a Swiss blood center and to investigate the influence of processing and donors’ characteristics.

**Methods:** The level of sO_2_ was measured in 1701 leukocyte-depleted RCCs derived from whole blood donations in both top–bottom (TB; component filtered, SAGM) and top–top (TT; whole blood filtration, PAGGSM) RCCs. The sO_2_ value was measured non-invasively through the PVC bag prior to storage by resonance Raman spectroscopy. Gender, age, blood type, hemoglobin level, and living altitude of donors, as well as process method and time-to-process were recorded.

**Results:** Overall, the sO_2_ exhibited a wide non-Gaussian distribution with a mean of 51.2 ± 18.5%. Use of top-top kits resulted in a 16% higher sO_2_ (*P* < 0.0001) than with top-bottom ones. Waiting time before processing only had a modest impact, but the blood processing itself reduced the sO_2_ by almost 12% (*P* < 0.0001). sO_2_ was also significantly affected by some donors’ characteristics. RCCs from men exhibited 25% higher sO_2_ (*P* < 0.0001) than those donated by women. Multivariate analysis revealed that the apparent correlation observed with hemoglobin level and age was actually due to multicollinearity with the sex variable. Finally, we noticed no significant differences across blood type but found that altitude of residence was associated with the sO_2_ (i.e., higher in higher living place).

**Conclusion:** These data confirm wide sO_2_ distribution in RCCs reported recently. The sO_2_ was impacted by the processing and also by donors’ characteristics such as the gender and the living altitude, but not by the hemoglobin level, blood group and donor age. This study provides new hints on the factors influencing red blood cells storage lesions, since they are known to be related to O_2_ content within the bags, giving clues to better process and to better store RCCs and therefore potentially improve the efficacy of transfusion.

## Introduction

Oxygen is required for aerobic organisms’ growth and survival. Indeed, oxidative phosphorylation, which consists in the reduction of O_2_ to H_2_O in the mitochondria, drives ATP production (Nelson and Cox, 2005). Transport of oxygen from the lungs to the tissues and of carbon dioxide the other way round is guaranteed by the red blood cells (RBCs). These cells, which are by far the most abundant host cell type within the human body (Greer et al., 2018), have specialized to optimize their gas carrier function. First, they lose their nucleus and most organelles during their maturation process so that their cytoplasm ends up mainly filled with hemoglobin [Hb; 92% of dry mass (Nemkov et al., 2018)]. Second, thanks to their biconcave shape that maximizes the area/volume ratio, these cells are capable of extensive deformation in order to cross the network of small capillaries for gas perfusion within tissues (Mohandas and Evans, 1994). Finally, to counteract the oxidative burden they are exposed to (because of their close interaction with O_2_ and the high levels of iron), RBCs possess powerful enzymatic and non-enzymatic antioxidant defenses (Cimen, 2008).

Transfusion of RBCs is indicated for patients suffering from anemia subsequently to a massive blood loss (e.g., trauma or surgery), a deficiency in RBC production (e.g., lack of folate, iron or erythropoietin), or an uncontrolled RBC destruction (e.g., sickle cell disease), etc. (European Committee on Blood Transfusion (CD-P-TS), 2020). RBCs for transfusion are stored in additive solution at 4°C in gas-permeable plastic blood bags. It is well known that during storage in such a non-physiological environment, the RBCs accumulate the so-called “RBC storage lesions” (D’Alessandro et al., 2015; Prudent et al., 2015; Bardyn et al., 2017; Yoshida et al., 2019). Whereas no consensus has been reached concerning a potential deleterious clinical impact, it is certain that the long-term stored RBCs exhibit metabolic (Bordbar et al., 2016; Paglia et al., 2016; D’Alessandro et al., 2017), oxidative (Kriebardis et al., 2006, 2007; Delobel et al., 2016), and functional alterations (Berezina et al., 2002; Rubin et al., 2012; Roussel et al., 2019) that reduce the quality of this blood product and could ultimately affect transfusion efficiency.

The extent of the lesions observed is variable among red cell concentrates (RCCs), which could indicate either a process- or a donor-dependency (Jordan et al., 2016; Tzounakas et al., 2016; Kanias et al., 2017; D’Alessandro et al., 2020). More particularly, it was observed that the initial oxygenation saturation (sO_2_) levels did widely vary among RCCs. Since oxidative stress is recognized as a major burden during storage, one could thus wonder about the effect of the basal sO_2_ levels. Yoshida et al. (2017) reported in 2017 a mean sO_2_ of 45.9 ± 17.5% with a 32.7–61.0 interquartile range (IQR). They also observed that high sO_2_ levels were associated with poorer quality of stored RBCs when looking at parameters such as ATP, hemolysis, metHb, oxidized lipids, and GSH/GSSG ratio.

The aim of the present study was to investigate the reasons (process- or donor-dependent) leading to such variable oxygen saturation levels. To do so, sO_2_ level was measured non-invasively in 1,701 RCCs and causal relationships with various variables (i.e., type of bags system, time-to-process, donors’ sex, age, Hb levels, blood type and living altitude) were assessed either by simple linear correlation between two factors or by multiple linear regression. This study aims at bringing new knowledge about the underlying causes of sO_2_ variability, to offer potential opportunities to rethink blood products donation, process and storage in order to improve the quality of the transfused blood products.

## Materials and Methods

### Data Collection

Level of sO_2_ was measured non-invasively through the PVC bag at 22°C prior to storage by Resonance Raman Spectroscopy on 1,701 RCCs. Among those 1,701 products, 112 blood bags were analyzed before and after processing. Data about blood processing (process method and time-to-process) and donors’ characteristics (sex, age, blood type, Hb level, and altitude of residence) were collected. The domicile corresponds to the address indicated in the donor database. Altitudes of municipalities (center of the town was considered) were obtained from Google Earth (version 9.3.111.2).

### Blood Products and Processing

Whole blood (WB; 450 ± 50 mL) was collected from healthy donors during November 2017 in different regions of Switzerland. Donors’ Hb level (g/L) was measured using the Hemo Control automated analyzer (EKF Diagnostics, Penarth, Cardiff, United Kingdom). This device uses the “gold standard” photometric azide metHb method (Vanzetti, 1966; Srivastava et al., 2014).

After collection in 63 mL citrate–phosphate–dextrose (CPD), the blood was processed following standard procedure in the preparation unit of Transfusion Interrégionale CRS. Leukoreduced RCCs (*n* = 1,701) derived from blood donations were either prepared in top-bottom bags system (TB; CQ32250, Fresenius-Kabi, Bad Homburg, Germany; *n* = 1366), or in top-top bags system (TT; FQE 6240LU, Macopharma, Tourcoing, France; *n* = 335). In TB bags system, blood components are first separated by centrifugation and are then filtered separately, in this case the additive solution used for RBC storage was saline-adenine-glucose-mannitol (SAGM). In TT bags system, WB is filtered before component separation, and phosphate-adenine-glucose-guanosine-saline-mannitol (PAGGSM) is used as additive solution. Of note, the measurements on the RCC units were non-invasive, therefore the analyzed RCC units were used for transfusion.

### Oximetry and Oxygen Saturation

Resonance Raman Spectroscopy was used to quantify the percentage of sO_2_ non-invasively. A Pendar probe (Pendar Microvascular Oximeter A3U11, Pendar Technologies, Cambridge, MA, United States) was placed on the surface of the blood bag. The oximeter recorded the resonance Raman spectrum of the RCC sample through the PVC bag. The bag was mixed carefully before analysis to homogenize its content because the 405 nm excitation light can only penetrate a few layers of RBCs behind the plastic surface. Difference in visible light absorption (i.e., color) between oxy-Hb (bright red, Raman line at 1380 cm^–1^) and deoxy-Hb (dark red, Raman line at 1355 cm^–1^) was used to estimate sO_2_, knowing that more than 95% of oxygen in a unit is bound to Hb inside RBCs.

### Data Analysis and Statistics

GraphPad Prism [version 8.4.2 (464), GraphPad Software, LLC., San Diego, CA, United States] was used for data presentation and statistical analyses. Minitab^®^ software (version 19.2020.1.0, Minitab, LLC., State College, PA, United States) was used for multiple regression analysis. To compare two datasets, the Mann–Whitney non-parametric *t*-test was used for unpaired data and the Wilcoxon signed rank test for matched-pairs. For multiple comparisons of unpaired groups, Kruskal–Wallis test with Dunn’s multiple comparison test was performed.

Simple linear regression was mainly used to evaluate the effect of a given parameter on the sO_2_ distribution. In this case, *F*-test was used to test the null hypothesis assuming that the overall slope is zero. Multiple linear regression (based on the least squares estimation technique) was also applied to model the relationship between sO_2_ and donors’ age, sex, Hb level, blood type, altitude of domicile, plus the time-to-process, and processing method.

For data presentation, Violin plots were used to show numeric distributions. Such representation is similar to box-and-whisker plots but includes a rotated kernel density plot on each side, which gives more details about the empirical distribution of the data. Lines at the median and quartiles were also plotted.

## Results

### Oxygen Saturation Within the Donor Population

**FIGURE 1:**
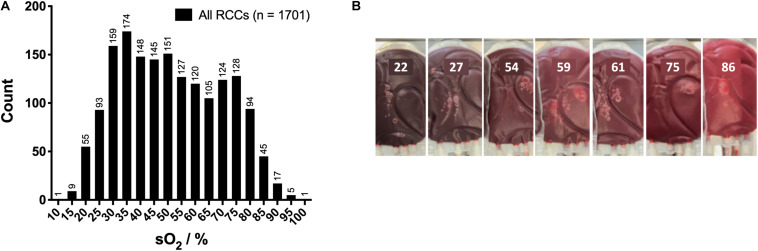
Hemoglobin oxygen saturation (sO_2_) within the red cell concentrates (RCCs). **(A)** Overall sO_2_ distribution in RCCs. **(B)** Color of RCCs and sO_2_ level.

Red cell concentrates (RCCs) from 1,701 healthy donors were analyzed in the framework of this study. Overall, the sO_2_ in RCCs exhibited a wide non-Gaussian distribution ranging from below 10% to above 90% with a mean of 51.2 ± 18.5%, a median at 50.0%, and an IQR of 32.0 (Figure 1A). Figure 1B is an example of RCC color as function of oxygen saturation level. It is the Hb that gives their red color to the RBCs, the tint of which depends on how many oxygen molecules are bound by the heme group. Poorly oxygenated RBCs (left pictures; higher level of deoxy-Hb) appear darker than well oxygenated RBCs (right pictures; higher level of oxy-Hb) that are scarlet.

### Effect of Blood Processing on Oxygen Saturation

**FIGURE 2:**
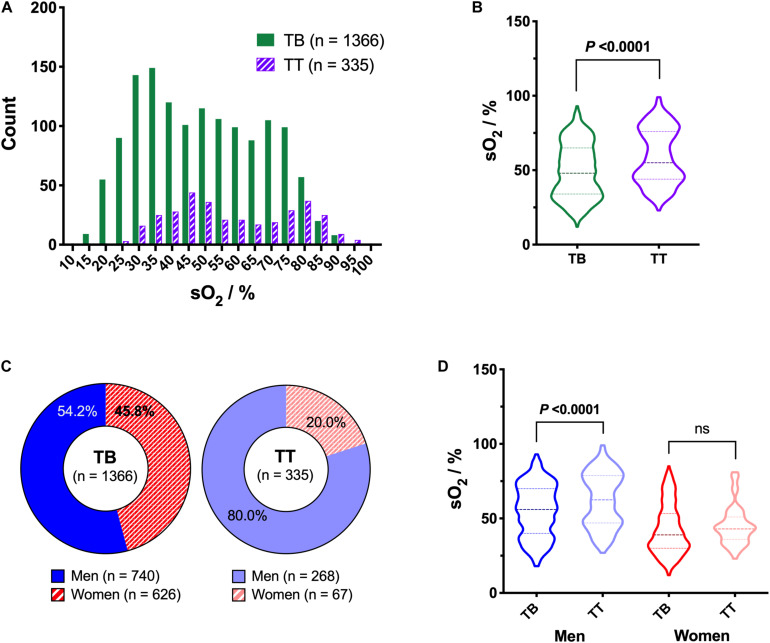
Impact of type of bags system on hemoglobin oxygen saturation (sO_2_), including effect of sex. **(A)** Histogram **(B)** and violin plot of sO_2_ distribution as function of the bags system used [i.e., top-bottom (TB) or top-top (TT)]. **(C)** Repartition of donors’ sex for the two bags systems. **(D)** Violin plot of sO_2_ as function of bags system and donors’ sex. *P* < 0.05 is considered significant, “ns” means non-significant.

The sO_2_ distribution depends on the bags system (Figure 2A). Here, 80.3% (*n* = 1,366) of the RCCs were prepared using TB bags system and 19.7% (*n* = 335) using TT bags system. TB processing showed a significantly lower sO_2_ than TT processing (*P* < 0.0001), with a mean of 49.4 ± 18.1% vs 58.9 ± 18.3%, respectively (Figure 2B). As presented in Figure 2C, sex repartition was not equal but close for TB RCCs with 54.2% men vs 45.8% women, whereas a majority (80.0%) of the TT RCCs were given by male donors. The effect of processing method on oxygen saturation level was in part influenced by the donors’ sex (Figure 2D). Indeed, the sO_2_ in TT remained significantly higher in the male group (*P* < 0.0001) with a mean of 62.3 ± 17.9% vs 55.2 ± 17.3% for TB RCCs. However, it was not significantly different in the female group (although a similar trend was observed), with a mean sO_2_ of 45.2 ± 13.0% in TT vs 42.5 ± 16.6% in TB RCCs.

**FIGURE 3:**
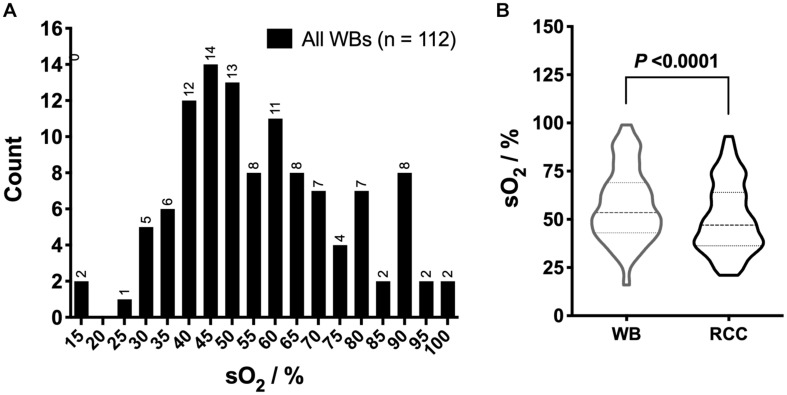
Hemoglobin oxygen saturation (sO_2_) in whole blood (WB) and red cell concentrates (RCCs) after processing. **(A)** Overall sO_2_ distribution in WB and **(B)** violin plot of sO_2_ distribution in WB bags and their paired RCCs. *P* < 0.05 is considered significant.

To evaluate the impact of blood processing, sO_2_ was measured in several bags before and after transformation (i.e., 112 WB units and their paired RCCs). Similar to the RCCs, the sO_2_ distribution within WB bags was wide (Figure 3A), with a mean of 57.3 ± 18.7%, a median at 53.5% and an IQR of 26.0. The processing from WB to RCC significantly decreased the mean sO_2_ to 50.6 ± 18.1% (*P* < 0.0001; Figure 3B).

**FIGURE 4:**
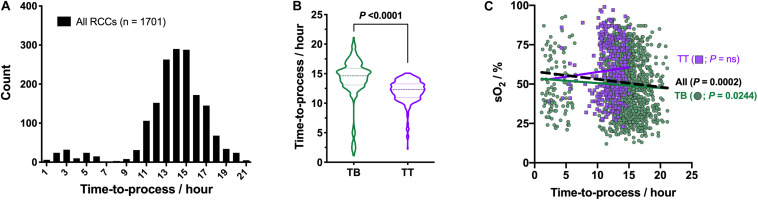
Effect of time-to-process on hemoglobin oxygen saturation (sO_2_). **(A)** Histogram of time-to-process distribution. **(B)** Violin plot of time-to-process for TB (n = 1366) and TT (*n* = 335) bags systems. **(C)** sO_2_ as function of time-to-process and type of bags system, with correlation analysis. Linear regressions: dashed black line for both bags systems and colored solid lines for each type of bag separately. *P* < 0.05 is considered significant, “ns” means non-significant.

The time-to-process that corresponds to the delay between the blood drawn and the separation steps lasted from 1.1 to 21.1 h, with a mean duration of 13.7 ± 3.4 h (Figure 4A). Of note, the waiting time was dependent on the type of bags system used for the processing (Figure 4B). The mean time-to-process was of 14.1 ± 3.6 h for TB vs 12.0 ± 1.9 h for TT products (*P* < 0.0001). Using simple linear regression analysis, it appears that the waiting time in WB had no repercussion on RBC oxygenation levels in RCCs when considering only TT RCCs, but they were negatively correlated when taking into account both bags systems (*P* = 0.0002, *R*^2^ = 0.008385; Figure 4C) or TB RCCs alone (*P* = 0.0244, *R*^2^ = 0.003708).

### Impact of Donors’ Characteristics on Oxygen Saturation

**FIGURE 5:**
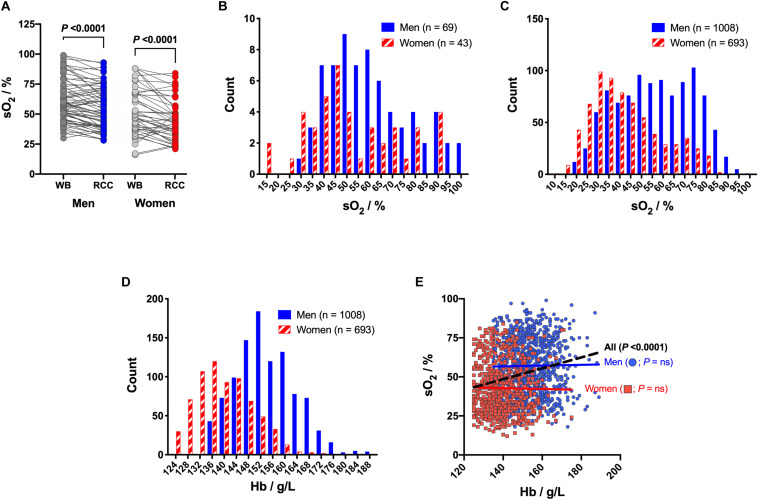
Hemoglobin oxygen saturation (sO_2_) in red cell concentrates (RCCs) and whole blood (WB) as function of donors’ sex and hemoglobin (Hb) level. **(A)** Evolution of sO_2_ levels during processing for men WB bags and paired men RCCs (*n* = 69) vs women WB bags and paired women RCCs (*n* = 43). **(B)** Histogram of sO_2_ distribution in WBs as function of donors’ sex. **(C)** Histogram of sO_2_ distribution in RCCs as function of donors’ sex. **(D)** Histogram of Hb level distribution as function of donors’ sex. **(E)** sO_2_ as function of Hb level and donors’ sex, with correlation analysis. Linear regressions: dashed black line for both sex and colored solid lines for each sex separately. *P* < 0.05 is considered significant, “ns” means non-significant.

Hemoglobin oxygen saturation was higher in blood products donated by men, both before and after processing (Figure 5A). Mean sO_2_ measured in male WB blood bags was of 60.4 ± 17.5% and of 52.3 ± 19.7% in the female ones (*P* = 0.0221; Figure 5B). In their paired RCCs, the sO_2_ dropped to 54.3 ± 17.7% (*P* < 0.0001) and 44.5 ± 17.2% (*P* < 0.0001), respectively. The effect of the sex variable on the sO_2_ was confirmed when considering all 1,701 RCCs (Figure 5C). Globally, RCCs from male donors exhibited higher sO_2_ than RCCs donated by women with means of 57.1 ± 17.7% vs 42.8 ± 16.3%, respectively (*P* < 0.0001). The sex difference in Hb levels, already described in the literature (Murphy, 2014; Greer et al., 2018), was also observed in the present donors cohort (Figure 5D), with a mean of 153.6 ± 9.9 g/L in men vs 139.4 ± 9.3 g/L Hb in women (*P* < 0.0001). Globally, the sO_2_ was weakly correlated to the Hb level taking into account both sexes together (*P* < 0.0001, *R*^2^ = 0.05027; Figure 5E). Nevertheless, it was not the case anymore when considering each sex independently; the level of sO_2_ is not a function of the concentration of Hb.

**FIGURE 6:**
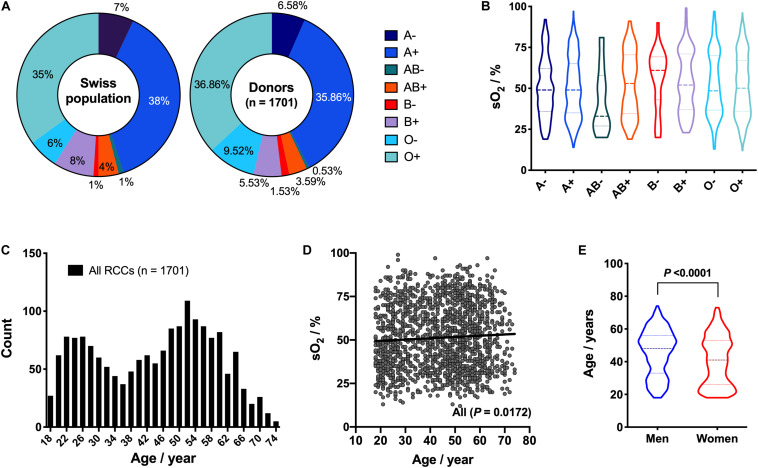
Effect of donors’ blood type and age on hemoglobin oxygen saturation (sO_2_). **(A)** Comparison of blood type repartition within the Swiss population and the donor cohort. **(B)** Violin plot of sO_2_ as function of donor’s blood type [i.e., A Rhesus - (*n* = 112), A Rhesus + (*n* = 610), AB Rhesus - (*n* = 9), AB Rhesus + (*n* = 61), B Rhesus - (*n* = 26), B Rhesus + (*n* = 94), O Rhesus - (*n* = 162), O Rhesus + (*n* = 627)]. **(C)** Histogram of donors’ age distribution. **(D)** sO_2_ as function of donors’ age, with correlation analysis. Solid line: linear regression. **(E)** Violin plot of donor’s age for men (*n* = 1,008) and women (*n* = 693). *P* < 0.05 is considered significant.

Blood type distribution within the 1,701 blood donors was similar to the average repartition observed in the Swiss population (Figure 6A). From statistical analysis, it appeared that the blood type was not an explanatory variable for the sO_2_ (Figure 6B). Of note, the small sample size for some blood types, such as AB Rhesus D- (*n* = 9) and B Rhesus D- (*n* = 26), could reduce the statistical power of the test. Donors’ age was comprised between 18 and 74 years, with a mean of 43.5 ± 14.6 years, with two local maxima at 26 and 52 years and a local minimum at 36 years (Figure 6C). Weak positive correlation was observed between donors’ age and sO_2_ (*P* = 0.0172, *R*^2^ = 0.003337; Figure 6D). When sexes were analyzed separately, no significant correlations with age were observed (data not shown). The observed effect is more related to sex than age. Indeed, male donors were generally older (*P* < 0.0001), with a mean age of 45.6 ± 13.9 years vs 40.4 ± 15.1 years for women (Figure 6E).

**FIGURE 7:**
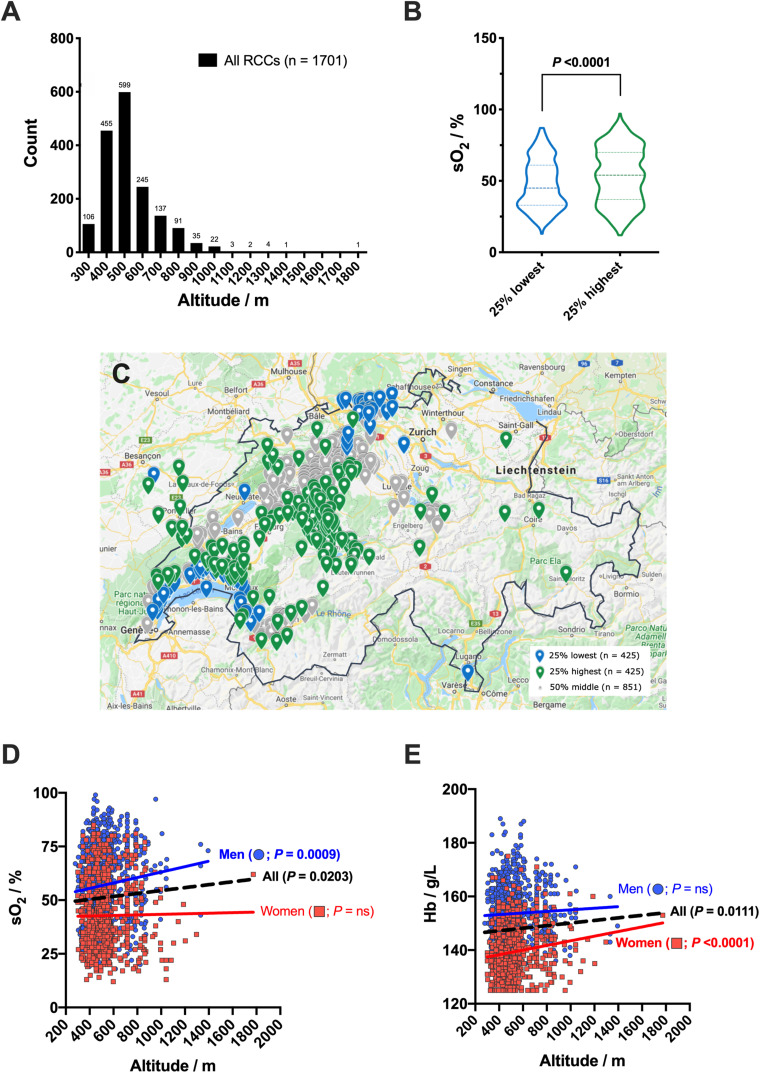
Altitude of donors’ domicile and hemoglobin oxygen saturation (sO_2_). **(A)** Histogram of donors’ domicile altitude distribution. **(B)** Violin plot of sO_2_ for the donors established in locations with the 25% highest (*n* = 425) vs the 25% lowest (*n* = 425) domicile altitude. **(C)** Geographical repartition of donors’ domiciles. **(D)** sO_2_ as function of domicile altitude and donors’ sex, with correlation analysis. **(E)** Hemoglobin (Hb) level as function of domicile altitude and donors’ sex, with correlation analysis. Linear regressions: dashed black line for both donors’ sex, and colored solid lines for men (*n* = 1,008) and women (*n* = 693) separately. *P* < 0.05 is considered significant, “ns” means non-significant.

The pool of donors studied came from various geographic areas in Switzerland (Figure 7C). This country being mountainous, the altitude of donors’ domicile ranged from 279 to 1773 m, with a mean at 528 ± 152 m and a median at 494 m (Figure 7A). Oxygen saturation was linked to the donors’ location. For example, the RCCs donated by the 25% of donors with the lowest vs the 25% with the highest domicile altitude (i.e., 382 ± 32 m vs 736 ± 148 m) exhibited a mean sO_2_ of 47.7 ± 17.0% vs 53.3 ± 19.3%, respectively (Figure 7B). Statistical comparison was significant (*P* < 0.0001). Of note, those two groups of 425 donors each had similar sex, age, Hb level and processing distributions, which confirms the influence of the living place. There was positive correlation between the sO_2_ and the residence altitude (*P* = 0.0203, *R*^2^ = 0.003166; Figure 7D), which was even more pronounced when considering only male donors (*P* = 0.0009, *R*^2^ = 0.01100). No correlation was observed in women. Interestingly, it appears that the Hb level and altitude of domicile were also correlated (*P* = 0.0111, *R*^2^ = 0.003792; Figure 7E), but was this time related to female donors (*P* < 0.0001, *R*^2^ = 0.02245) and not to men.

### Multivariate Analysis of Donors’ Characteristics and Processing Parameters With Oxygen Saturation

Using Minitab^®^ software, multiple linear modeling was applied to test the causal relationship between predictors (i.e., time-to-process, bags system, donors’ sex, Hb level, age, blood type, altitude of domicile) and the oxygen saturation within RCCs. To do so, least squares regression was applied. Detailed results of the analysis are available in Supplementary Material.

Multivariate analysis confirmed the association between sO_2_ levels and the sex (*P* < 0.0001), the type of bags system (*P* < 0.0001), and to less extent with the living altitude (*P* = 0.004) and the time-to-process (*P* = 0.042), but not with the age (*P* = 0.777), Hb concentration (*P* = 0.892) nor the blood type (*P* = 0.600).

## Discussion

Distribution of sO_2_ of the 1701 RCC units was similar to the one described by Yoshida et al. (2017), who measured it by co-oximetry using an ABL90 Flex blood gas analyzer (Radiometer, Copenhagen, Denmark). Similarly, the wide data distribution observed did not pass the normal distribution tests applied (i.e., Anderson-Darling, D’Agostino & Pearson, Shapiro–Wilk and Kolmogorov–Smirnov tests). An observed wide distribution of venous blood was already described in 1938 by Ancel Keys. He measured an average saturation of 68.2% in blood from the arm veins of 63 healthy subjects at rest, with values ranging from 29 to 89% (Keys, 1938). Whereas arterial blood oxygen saturation is generally above 95%, venous sO_2_ levels are today considered normal for values comprised between 55 and 80%, depending on the source of information. In clinics, venous oxygen saturation is generally obtained from internal jugular of subclavian catheters or less often from pulmonary artery catheter measurements. In such case, the sO_2_ levels represent the leftover of what has not been used by the tissues and organs. During blood donation, phlebotomy is made in donors’ arm veins (Dhingra et al., 2010). Therefore, sO_2_ values measured in RCC bags could not be entirely representative of the blood oxygenation state within the body, but variation could also be linked to changes occurring after blood collection, and therefore be related to the intrinsic affinity of Hb for oxygen, as well as the preparation procedure.

In the present study, sO_2_ was also measured in 112 WB units and again in their paired RCCs after blood component separation, revealing that the process reduced oxygen saturation by nearly 12%. Moreover, the sO_2_ level was negatively correlated with the time-to-process.

The type of bags system used for processing also had a significant impact. With the TB bags system, blood components are first separated and only the RBCs are filtrated and stored for up to 42 days in SAGM additive solution at 4°C. In this process, WB units were rested at room temperature for 14.1 ± 3.6 h before RBCs were separated from the plasma and the buffy coat (used for fabrication of platelet concentrates). With TT bags system, blood components are extracted by centrifugation after WB filtration and RBCs resuspended in PAGGSM additive solution usually within 12.0 ± 1.9 h to be stored up to 49 days at 4°C. TT plasma can be used for transfusion, and in such case, male donors are preferentially selected in order to limit the risk of adverse effect associated with HLA antibodies (that may occur during pregnancy) (Pandey and Vyas, 2012). It explains why proportion of TT RCCs donated by men was five times higher than those donated by women. However, although strongly impacted by the donors’ sex, correlation as well as multivariate analysis highlighted that the type of bag was also significantly contributing to explain the sO_2_ levels within the RCCs (at least for male donors). Since the time-to-process was negatively correlated with the sO_2_ level, oxygen consumption by platelets and leukocytes likely overcame the O_2_ influx via PVC bag during the room temperature hold, thereby resulting in lower sO_2_. The differences in bag and/or filter composition, and/or layer thickness of the plastic blood bag might also be a reason of the observed differences between the two types of kits.

Large scale study was also the opportunity to make some general observations about the socio-demographic characteristics of the blood donor cohort and more particularly about the influence of age. Donation prevalence was the highest at 52 years with a local maximum at 24 years and a local minimum at 36 years. Decrease of blood donation at this age is very likely related to the age of the youngest child (Burgdorf et al., 2017). Indeed, women who are or were pregnant are not allowed to give blood, with a deferral period of 12 months from delivery. The same trend was observed in male donors, probably because of the “rush hour of life,” a phase in which life’s major tasks are concentrated (children, career, caring for one’s parents). Overall, the mean age of donation for women was 5.2 years lower than men, which introduced a bias in the correlation analysis between the age and the sO_2_ level. Absence of contribution of the age variable was confirmed by multivariate analysis. Similarly, the blood type did not impact the oxygen level.

Female donations represented 40.7% of all blood products analyzed in this study. The sex was the variable demonstrating the strongest association with the sO_2_, with an *F*-value of 156.53. As expected, hemoglobin concentration was also strongly correlated with the donors’ sex, women having an approximatively 9% lower Hb level. Higher sO_2_ level in men can be explained by a stronger Hb-O_2_ affinity compared to women (Humpeler and Amor, 1973; Greer et al., 2018; Balcerek et al., 2020). Release of O_2_ is favored by several factors such as acidosis, increased CO_2_ levels and temperature, as well as elevated 2,3-Diphosphoglycerate (2,3-DPG) concentration. The latter was shown to be higher (2 μmol/gHb) in women but only after puberty and before menopause, suggesting that sex hormones – and particularly estrogens – are implicated in the O_2_ transport capacity (Humpeler et al., 1989). Among other roles, the sexual hormones estrogens and androgens control vascular resistance. They modulate the Fåhraeus effect which corresponds to the fall of hematocrit in vessels below 300 μm in diameter (Murphy, 2014). The female hormones estrogens being vasodilators, the hematocrit of women in their microvasculature is consequently higher. The opposite effect is driven by male androgens that promote vasoconstriction, notably in the kidney, reducing oxygen delivery to the juxtaglomerular apparatus, triggering an increase of the red cell mass by erythropoiesis. Higher Hb level in men compensates lower release of oxygen within tissue because of the high Hb-O_2_ affinity, ensuring an adequate cellular oxygen availability.

We speculated that the bimodal distribution of sO_2_ level in women could be attributed to changes in the balance of the sex hormones at menopause. Indeed, during this period (around 51 years in average in Switzerland), the estrogen concentration in women decreases to a level similar to that of men. Surprisingly, no correlation was found between women’s age and RCC sO_2_ level. However, a significant difference (*P* = 0.0291) was found in the age of women whose RCC had less than 60% sO_2_ versus those having 60% sO_2_ or more (i.e., 39.8 versus 42.9 years old, respectively).

Because no additional questionnaire was filled in the present study, the location used to determine the altitude of the donors’ domicile was the one indicated in our database. Therefore, it cannot be excluded that some donors spend time somewhere else (e.g., workplace, students at university) at a lower or a higher altitude, introducing a bias in the analysis. Nevertheless, based on the data available, it seems that the altitude did impact the blood oxygenation, especially when comparing the 25% donors having the lowest and the 25% the highest altitudes of domicile. Weak correlation was obtained between the altitude and sO_2_ or between the altitude and Hb variable. Nevertheless, stronger effect would be observed if the range of living altitude was broader. Indeed, the effect of the altitude on Hb levels is well known. Magnitude of difference found is also impacted by the ethnic group and the sex (Gassmann et al., 2019).

In the present study, the effect on sO_2_ level of other factors such as the donors’ physiological (e.g., height, muscular mass, history of hypertension, etc.) or biological characteristics (e.g., 2,3-DPG and hormone levels, as well as ethnicity); degree of stress and oxygenation (e.g., room ventilation) during donation; environmental (e.g., pollution exposure); or lifestyle (e.g., physical activity, sleeping, eating, drinking and smoking habits) cannot be excluded and would be interesting to further explore.

Since RCCs are known to get oxygenated during hypothermic storage (Yoshida et al., 2017) and knowing that storage lesions are driven by oxidative stress (D’Alessandro et al., 2015; Bardyn et al., 2018), storage of high sO_2_-level units might accelerate the storage lesions. Indeed, it is known that the cold storage under anaerobic or reduced O_2_ content reduces the lesions to RBCs (Yoshida et al., 2007; Burns et al., 2016; Prudent et al., 2016). Of interest, it was reported that the hemolysis rates are lower in RCCs from females (Jordan et al., 2016). Hence, the overall quality of RCCs could be significantly improved by storage in an O_2_ controlled environment. Selection of the donors or modification in the donation conditions (e.g., in hypoxic conditions or during a physical activity) and processing (e.g., use of bags system pre-filled with inert gas or under vacuum) could also be investigated.

## Conclusion

Independently of the level of Hb, the sO_2_ was influenced by the processing: type of bags system and time-to-process, and by several donors’ characteristics: such as the sex and living place (with a positive correlation with the altitude). However, none of the examined factors significantly affected the overall wide breadth of the distribution.

The present study brings information on the impact of donors’ characteristics and blood collection/processing methodologies on stored RBCs, which could impact the quality of transfused RBCs and potentially affect the patient outcome. The experiments on a large number of RCCs confirm the wide sO_2_ distribution from venous blood reported recently (Yoshida et al., 2017). Additional investigations (including a detailed questionnaire, medical history and other clinical evaluations of donors) would enable further investigations on the origin of these differences. The correction of these variations by *ad hoc* processing could be an option to improve the storage of RBCs and thus the quality of the RCCs for transfusion.

## Data Availability Statement

The raw data supporting the conclusions of this article will be made available by the authors, without undue reservation.

## Ethics Statement

The studies involving human participants were reviewed and approved by Institutional Review Board of Transfusion Interrégionale CRS. Written informed consent for participation was not required for this study in accordance with the national legislation and the institutional requirements.

## Author Contributions

TY and MP designed the study. ND provided the blood bags. AM and TY measured the sO_2_ levels in the WB and RBC bags. MP and MB treated and interpreted the data. MB performed statistical analysis, prepared the figures and wrote the draft of the manuscript. All authors contributed to the final version of the article and approved it for publication.

## Conflict of Interest

MP and MB have received financial support from Hemanext. AD and TY are employees from Hemanext. The remaining authors declare that the research was conducted in the absence of any commercial or financial relationships that could be construed as a potential conflict of interest.
