# Supplementary material for: Oxygen in Red Blood Cell Concentrates: Influence of Donors’ Characteristics and Blood Processing
Source: Front Physiol. 2020 Dec 23;11:616457. doi: 10.3389/fphys.2020.616457 (PMC7786264; doi:10.3389/fphys.2020.616457)
Supplement: Supplementary file 1 [file Data_Sheet_1.PDF]

Supplementary Material

1 Multivariate Analysis of Donors' Characteristics and Processing Parameters Associated with Oxygen Saturation within Red Cell Concentrates

Detailed results of the multivariate analysis performed using Minitab® software are presented below: with the regression equation (Equation 1), the analysis of variance table (Table 1), the model summary table (Table 2) and the coefficients table (Table 3).

1.1 Equation 1. Regression Equation

$$
\begin{aligned}
sO_2(\%) = {} & 40.73 - 0.0081\,Age\,(year) - 0.0058\,[Hemoglobin]\left(\frac{g}{L}\right) \\
& - 0.252\,Time_{to_{process}}(hour) + 0.00779\,Altitude\,(m) + 0.0\,ABO\,(A\,Rhesus_-) \\
& + 1.63\,ABO\,(A\,Rhesus_+) + 1.01\,ABO\,(AB\,Rhesus_-) + 3.16\,ABO\,(AB\,Rhesus_+) \\
& + 6.31\,ABO\,(B\,Rhesus_-) + 4.23\,ABO\,(B\,Rhesus_+) + 2.29\,ABO\,(O\,Rhesus_-) \\
& + 2.04\,ABO\,(O\,Rhesus_+) + 0.0\,Bags\ system\,(Top_{Bottom}) \\
& + 5.53\,Bags\ system\,\left(Top_{Top}\right) + 0.0\,Sex\,(Female) + 13.42\,Sex\,(Male)
\end{aligned}
$$

1.2 Table 1. Analysis of Variance

Source	DF	Adjusted SS	Adjusted MS	F-Value	P-Value
**Regression**	13	98317	7562.9	26.31	0
**Age / year**	1	23	23.1	0.08	0.777
**[Hemoglobin] / g/L**	1	5	5.3	0.02	0.892
**Time-to-process / hour**	1	1185	1185	4.12	0.042
**Altitude / m**	1	2354	2354.1	8.19	0.004
**Blood type**	7	1579	225.5	0.78	0.6
**Bags system**	1	7390	7389.9	25.7	0
**Sex**	1	45105	45105.2	156.89	0

DF: degrees of freedom; SS: sum of squares; MS: mean squares.

1.3 Table 2. Model Summary

S	R^2	R^2 (adjusted)	R^2 (predicted)
16.9557	16.85%	16.21%	15.41%

1.4 Table 3. Coefficients

Term	Coefficient	SE Coefficient	T-Value	P-Value	VIF
**Constant**	40.73	6.64	6.13	0	
**Age / year**	-0.0081	0.0287	-0.28	0.777	1.04
**[Hemoglobin] / g/L**	-0.0058	0.0429	-0.14	0.892	1.55
**Time-to-process / hour**	-0.252	0.124	-2.03	0.042	1.06
**Altitude / m**	0.00779	0.00272	2.86	0.004	1.02
**Blood type**					
**A pos**	1.63	1.75	0.93	0.352	4.15
**AB neg**	1.01	5.9	0.17	0.865	1.09
**AB pos**	3.16	2.7	1.17	0.243	1.5
**B neg**	6.31	3.69	1.71	0.088	1.21
**B pos**	4.23	2.38	1.78	0.076	1.74
**O neg**	2.29	2.09	1.1	0.274	2.22
**O pos**	2.04	1.74	1.17	0.242	4.19
**Bags system**					
**Top-top**	5.53	1.09	5.07	0	1.11
**Sex**					
**M**	13.42	1.07	12.53	0	1.64

SE: standard error; VIF: variance inflation factor.
